# Antioxidants to Supplement or Not to Supplement That Is the Question

**DOI:** 10.3390/antiox1010001

**Published:** 2012-09-25

**Authors:** Stanley Omaye, Nabil Elsayed

**Affiliations:** 1 Department of Agriculture, Nutrition and Veterinary Sciences, University of Nevada, 1664 North Virginia Street, Reno, NV 89557, USA; 2 SUNY Down State Medical Center, Brooklyn, NY 11203, USA

Antioxidants, whether from diet or pharmacological supplementation, gained significant popularity among scientists and lay public in recent years, and was claimed to protect or treat numerous ailments. For decades, it has been recognized in the biomedical field that a balance exists between prooxidants and antioxidants in biological systems, and disturbance of that balance in favor of the first can result in “oxidative stress” [1]. Many factors were identified to disturb this balance leading to free radical formation thought to be associated with physiological and pathophysiological phenomena, such as aging, declining immunity, and many diseases including carcinogenesis, cardiovascular diseases (CVD), metabolic and inflammatory alterations, age-related macular degeneration (AMD), and osteoporosis. Moreover, environmental events such as inhalation of oxidant air pollutants [2], and exposure to explosion-generated shock waves [3] were proposed to be associated with free radicals generation, and antioxidant depletion. Consequently, the following question was posed: Can antioxidant supplementation protect or prevent from disease or environmental threats? In animals, the results obtained from experimental studies indicated that supplementation with antioxidants either at pharmacologic high-doses or physiologic nutritional doses reduced the risk of disease, protected from environmental insults, and restored depleted endogenous antioxidants. However, in humans, the results were not as clear [4,5,6,7,8,9]. Possibly, due to the interactions of many confounding factors and individual life styles such as smoking and obesity. Today, inconclusive evidence exists in support of long-term, high-dose antioxidant supplementation for protection or prevention of disease, and some epidemiological studies even suggested that supplementation not only fails to produce tangible improvement, but can produce opposite results and death. For example, in 2009, the Physicians Health Study II concluded that vitamin C and E failed to prevent heart attacks, strokes, prostate and total cancer, and death from CVD, in men [10,11]. Antioxidant supplements also did not prevent fatty buildup in the arteries and interfered with cholesterol-lowering drugs. In contrast, a review of dietary polyphenol conducted in 2009 [12] showed that diets rich in antioxidants, unlike pharmacological supplements, successfully protected from heart disease. The list includes carotene, vitamins E, C, selenium, and flavonoids, among others. These findings lead to another question: Is there a threshold of antioxidant supplements above or below which the human body will react negatively to oxidative stress? 

Few would disagree with the importance of fruits and vegetable in a healthy diet and the antioxidant contribution they provide as bioactive compounds. Since fruits and vegetable contain mixtures of antioxidants, a number of questions come to mind: Is the combination of antioxidants more beneficial than individual antioxidants, and can we define the composition of such mixtures with respect to optimal concentrations for human health? Under what physiological or pathological situations will antioxidants affect health adversely? Can excess antioxidants compromise normal body functions where oxidative stress plays a crucial role to health similar to the oxidative burst in phagocytosis? Do dietary antioxidants provide supplementation at an optimum threshold? And therefore, a healthy diet containing fruits and vegetables coupled with moderate exercise would provide a more effective means of protection or prevention from disease or slowing down of aging better than pharmacologic supplementation would do [13,14]? Finally, before we close the book on antioxidant supplementation, there is no doubt that more research remains to be done, and we invite all researchers in the field to provide results of their studies as well as their comments and opinions for or against antioxidant supplementation in this journal. 
